# The energy-health-environment nexus: assessing the transboundary impacts of coal-fired power plants

**DOI:** 10.3389/fpubh.2025.1581460

**Published:** 2025-07-31

**Authors:** Raja Dhar, Sayantan Sarkar, Chen Luo, Megha Anand, Shyamasree Dasgupta, Ujjwal Neogi, Apostolos Bossios, Rausan Zamir, Mohammad Shoeb, Joyanto Routh

**Affiliations:** ^1^Department of Pulmonology, Calcutta Medical Research Institute, Kolkata, India; ^2^School of Civil and Environmental Engineering, Indian Institute of Technology Mandi, Mandi, Himachal Pradesh, India; ^3^Department of Thematic Studies, Linköping University, Linköping, Sweden; ^4^School of Humanities and Social Sciences, Indian Institute of Technology Mandi, Mandi, Himachal Pradesh, India; ^5^Department of Laboratory Medicine, Karolinska Institute, Stockholm, Sweden; ^6^Division for Lung and Airway Research, Institute of Environmental Medicine, Karolinska Institutet, and Karolinska Severe Asthma Center, Department of Respiratory Medicine and Allergy, Karolinska University Hospital, and Center for Molecular Medicine, Karolinska University Hospital, Stockholm, Sweden; ^7^Department of Chemistry, Rajshahi University, Rajshahi, Bangladesh; ^8^Department of Chemistry, University of Dhaka, Dhaka, Bangladesh

**Keywords:** coal, power plant emissions, transboundary pollution, lung health, socioeconomic status

## Abstract

Point source emissions from large coal-fired power plants are pivotal in the energy-health-environment nexus, impacting energy security, air quality, and public health outcomes. Despite this, there is a lack of interdisciplinary prospective studies focusing on the effects of power plant emissions on the population residing downwind. To address this gap, a comprehensive, multicenter, interdisciplinary study on a transboundary scale (India and Bangladesh) has been launched, which includes modeling power plant emissions, seasonal collection of particulate matter and its chemical analysis, socioeconomic surveys of the case (downwind) and control (upwind) populations, lung health assessments, and transcriptomic analyses of blood samples. The outcomes will provide quantitative estimates of power plant impacts on air quality, lung health, and blood markers associated with pulmonary complications. This approach comprehensively assesses the population health impacts of power plant emissions. Moreover, by conducting lung tests in patients on-site, the health team captures an actual snapshot of air pollution. The study establishes causality between power plant emissions, particulate matter characteristics, and population exposure levels while accounting for social, demographic, and economic factors, the study establishes. By integrating diverse techniques, quantitative and qualitative methods, and perspectives, this study aims to enhance scientific understanding of the health and environmental risks and the socioeconomic burden associated with coal-based energy generation in developing countries.

## Highlights

- The proposed framework assesses the impacts of coal-fired power plants on air quality and community health.
- Health and socioeconomic surveys offer insights into the severity of medical and economic hardships.
- Particulate matter characterization and toxicological assessments are crucial for evaluating causality and respiratory disease.
- Air quality, health, and economic concerns could drive policies that reduce coal consumption and use clean energy.

## 1 Introduction

Coal-fired thermal power plants (TPPs) generate large amounts of particulate matter (PM), toxic and greenhouse gases (NOx, SO_2_, CO, O_3_, and CO_2_), various potentially toxic elements (As, Cd, Cu, Pb, Hg, and Zn), and toxic organic compounds (polycyclic aromatic hydrocarbons, dioxins, and quinones). These emissions pose multifaceted environmental, health, and social challenges. Harmful emissions released from tall stacks in TPPs travel afar, propelled by local or prevailing winds. There are no transboundary limits, and these emissions follow the atmospheric circulation patterns, resulting in a pollution footprint that stretches for hundreds of kilometers. Prolonged exposure and inhalation of these toxic pollutants, especially in older people with comorbid conditions and young children, could quickly worsen symptoms, leading to a steady deterioration in health or, even worse, cause fatalities. In particular, the emissions can cause respiratory ailments by contributing to the development or exacerbation of asthma and allergic sensitization, as well as an increased risk of the onset or worsening of COPD (chronic obstructive pulmonary disease) (1–3).

Recent estimates propose that air pollution contributes to 8.34 million excess deaths annually (4). Of these, around 5.13 million casualties are linked to ambient air pollution from fossil fuel use, with coal combustion emerging as a significant contributor to this staggering mortality (4, 5). While the exact number of TPP-related fatalities is unknown, widespread increases in TPP construction to meet the surging energy demands and deteriorating air quality in many densely populated regions highlight the severity of this global issue. It is no surprise that people living near the TPPs have exhibited increased symptoms of coughing (6), pathophysiological changes shown by oxidative stress (7), and respiratory diseases such as asthma, bronchitis, and COPD (8). Besides this, complications such as higher likelihoods of pre-term delivery and lower birth weight (9, 10), children developing low IQ (11, 12), and diminished life expectancy in adults have also been reported (5).

The International Energy Agency states that India is the world's second-largest country in terms of production and consumption of coal. India, a rising economy, heavily relies on coal to meet >70% of its soaring energy demands for a population exceeding 1.4 billion. This trend will likely continue into the foreseeable future, resulting in a further increase in coal consumption and aggravated health impacts stemming from PM exposure compared to the previous levels (13, 14). The respiratory health of people exposed to TPP emissions will likely be affected further due to simultaneous exposure to air pollution caused by biomass burning for cooking or household heating, smoking, vehicular traffic, and other environmental or lifestyle-related choices. Hence, it is imperative to accurately evaluate the effects of TPP emissions while factoring in activities like indoor household pollution from biomass burning and smoking that could mask the impacts of TPP emissions on respiratory health. A clear understanding of the different sources and impacts of TPP emissions is crucial and a prerequisite for developing informed strategies to mitigate potential health risks and ensure the community's wellbeing. Therefore, ongoing and future studies should unequivocally establish the provenance of these emissions and comprehend how exposure to TPP emissions impacts people's health, including the biological alterations they induce.

## 2 Methodological framework

An interdisciplinary team leads the investigation, including expertise in environmental chemistry, aerosol modeling, pulmonology, environmental economics, and systems biology. It is structured as a multinational, multicenter prospective study aiming to clarify the relationship between TPP emissions and PM exposure to enhance the scientific understanding of TPP-related health risks, environmental impacts, and socioeconomic costs. We hypothesize that the manifestation and severity of health issues resulting from exposure to TPP emissions exhibit variations based on the chemical composition of PM, atmospheric dispersion of emissions related to seasonality, socioeconomic conditions, and chronic exposure effects.

The prolonged exposure to emissions may accelerate lung aging, resulting in the early onset of respiratory complications (15). Hence, the focus of this study revolves around evaluating respiratory health in subjects residing near TPPs. We also incorporate information on cooking fuel used during the questionnaire survey to elucidate the interplay between indoor and outdoor air pollution. The study is based on the Farakka National Thermal Power Corporation plant in the Murshidabad district of West Bengal (India), a coal-fired mega TPP located around 20 km west of the India-Bangladesh border. The Farakka power plant (24°46′N, 87°53′E) is a “super” category unit capable of generating 2,100 MW that has been operational since 1986. Coal used in the TPP is primarily mined in the nearby Raniganj coal mines, producing a variety of low-grade coal with high ash content (16). Fly ash produced by the TPP is stored on-site in ash ponds, and part of it is used for making bricks or in the construction industry.

We systematically tackle the challenges outlined here to generate valuable data and enhance our understanding of:

a) how can we establish discernible respiratory effects in the community attributed to point source emissions, such as those from a TPP, while eliminating other contributing factors (e.g., biomass burning, smoking, and industrial or traffic emissions)?
b) how do socioeconomic factors and demographic status influence exposure to PM emissions and respiratory health outcomes?
c) how does transboundary dispersion of aerosols from TPPs by coal-dependent countries create a “historical legacy” of air pollution and policy issues affecting economically disadvantaged nations?

## 3 Discussion

**Figure 1:**
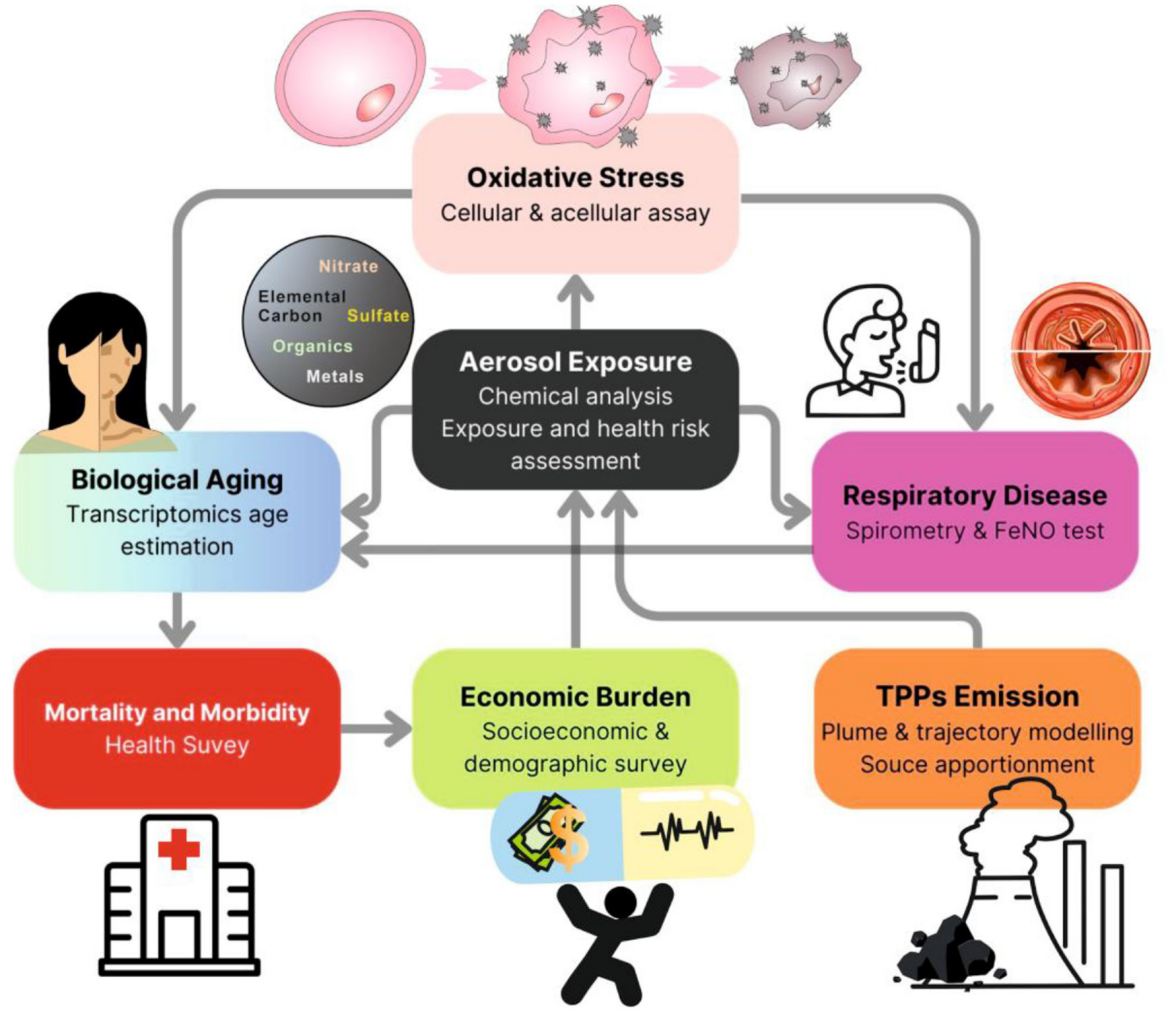
A flow chart indicating the different investigations outlined in this study to delineate the impact of coal-fired thermal power plant emissions on the environment, human health, and society.

The proposed framework (items 1–4, see below) is being pursued to align with our intended goals. The comprehensive framework adopted in this prospective study includes socioeconomic surveys, aerosol sampling, spirometry, and fractional exhaled nitric oxide (FeNO) in the subjects on a seasonal basis (winter, monsoon, and post-monsoon seasons; Figure 1). Blood sampling was conducted only once. Adult males/females (18–80 years) were recruited for the socioeconomic surveys, lung functionality testing, and blood collection. Only a subset of the adult population surveyed was considered for lung function tests and blood collection. All participants provided written informed consent, voluntarily agreeing to contribute to the study.

The strategic framework will guide environmental and clinical studies on how point source emissions, particulate matter characteristics, and socioeconomic conditions collectively impact respiratory health. It will guide researchers designing interdisciplinary studies to gather qualitative and quantitative data, enabling the interpretation of complex environmental, health, societal, and policy challenges. By doing so, this framework will develop strategies that enhance advanced preparedness to tackle air pollution, address shared challenges, and foster mutual benefits and knowledge-sharing within communities.

### 3.1 Air pollution modeling and site selection

**Figure 2:**
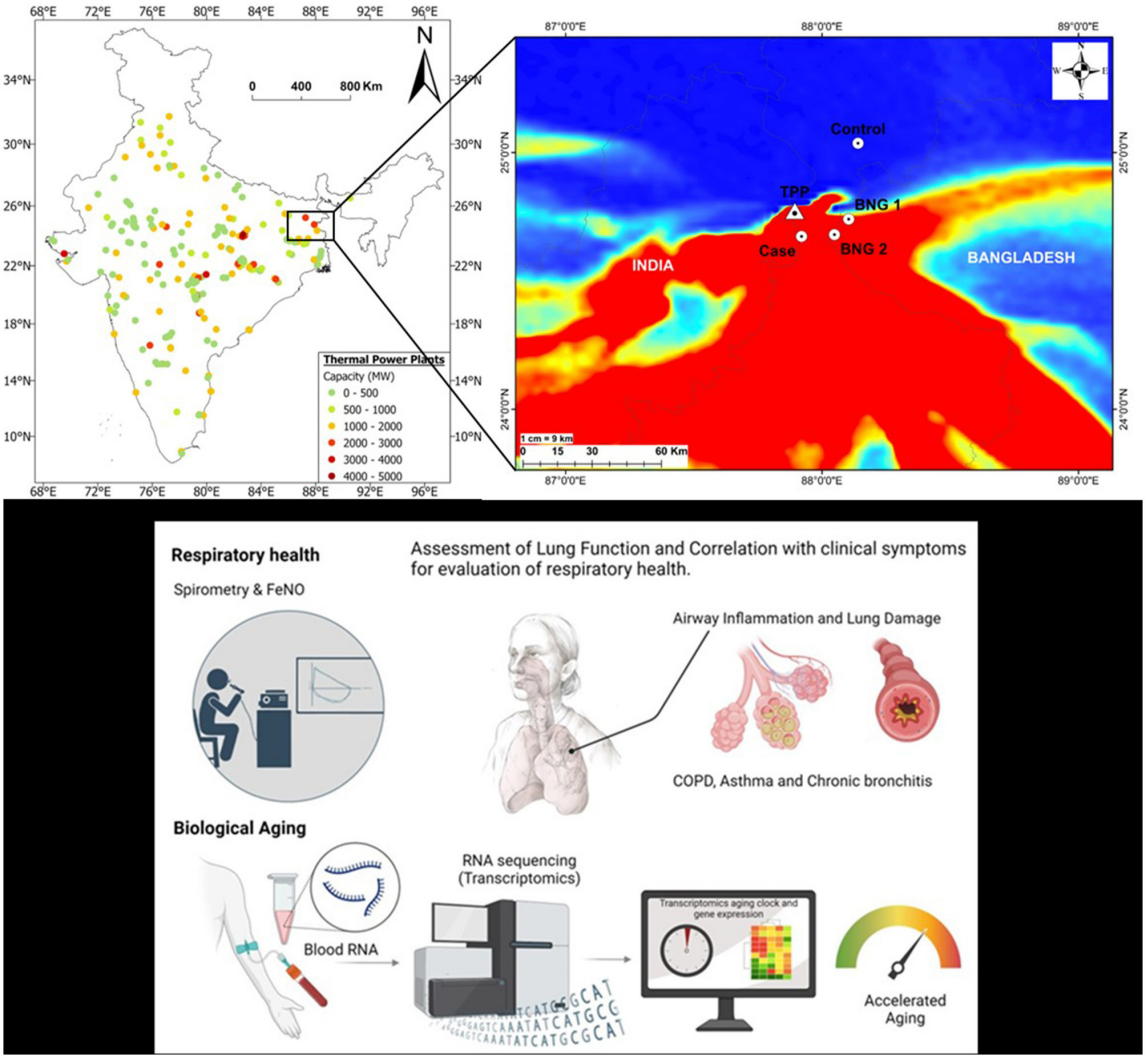
**(A)** Location of coal-fired thermal power plants in India and their capacity (28). The inset shows the transport of the plume across the Indo-Bangladesh border (TPP Farakka Thermal Power Plant); *Case* (affected downwind site in Murshidabad district, West Bengal), *Control* (background upwind location in Malda district, West Bengal); *BNG 1* and *BNG 2* (sites investigated in Rajshahi district in Bangladesh). **(B)** Spirometry and FeNo will be used to address lung function and airway inflammation, which, together with clinical data, will help us to diagnose the presence of airway diseases, such as asthma, COPD, and chronic bronchitis. **(C)** RNA will be isolated from blood, following transcriptomics analysis to evaluate lung aging. The spirometry image is from https://www.cleanpng.com/users/@carla3rd.html, personal use, and lung image was created with bioRender.

The first step in the study includes identifying the *Case* (site most affected by TPP emissions) and *the Control group* (site mostly unaffected by TPP emissions; background). We used numerical modeling to estimate how PM emitted by the TPP disperses. The FLEXPART dispersion model was used to calculate the spatial distribution of sulfate as a proxy for TPP emissions by using 3 km × 3 km meteorological data derived from the Advanced Weather Research and Forecasting (WRF) model. Based on the dispersion results, the *Case* and *Control* sites (~11 km downwind and 30 km upwind from the TPP, respectively) were selected (Figure 2). The results indicate that the PM plume from the Indian side crosses the international border into neighboring Bangladesh and contributes to the ambient PM levels. Hence, we selected two additional sites in Rajshahi, Bangladesh, strategically located ~25 km downwind from the Farakka TPP, to track the transboundary impacts of emissions.

### 3.2 Estimating costs of chronic exposure through socioeconomic surveys

Household surveys were undertaken at the *Case* and *Control* sites in India and Bangladesh based on simple random sampling to assess the socioeconomic conditions, demographic characteristics, and the cost of health-related externalities. The sites have a predominantly stable, non-transient population (based on the state voter's list from the local government) and experience minimal traffic congestion at any time of the day.

The sample size for the survey was calculated based on the formula:


(1) $$\begin{array}{l} Sample\;size\;\left(n\right)\;=\frac{\frac{z^{2}\times p\;\left(1-p\right)}{e^{2}}}{1+\left[\frac{z^{2}\;\times p\;\left(1-p\right)}{e^{2}N}\right]} \end{array}$$

where *z* is the *z*-value corresponding to the desired confidence level, *p* is the estimated proportion of the characteristic of interest, *q* = (1 – *p*), *N* is the known population size, and *e* is the permissible margin of error. The sample size was calculated on a 95% confidence interval and a 5% margin of error. Based on India's population and average family size (~4.5), ~100 households were selected at each site, representing ~401 individuals at the control site and ~385 at the case site. In Bangladesh, 50 households were surveyed, representing ~200 individuals, given an average family size of ~4.1. To accurately capture the population with prolonged exposure to TPP emissions, only people residing in the area for 10 years or more are included in the study. While the selection of sample size in this manner determines the power of the test, Cohen's *D* and Pearson's Correlation could be deployed to check the effect size.

A questionnaire (Table 1) was designed to systematically collect data on: 1) socioeconomic and demographic characteristics, 2) frequency and type(s) of respiratory ailments, 3) frequency of hospital visits due to respiratory issues, 4) household costs associated with illness, loss of productivity, and wages, 5) smoking and cooking practices, and 6) morbidity and mortality frequency. Following ongoing detailed statistical tests, these datasets will help us understand the differences in impacts and health repercussions stemming from exposure to PM emissions between the different locations, considering the observed variations across income groups, age, gender, and livelihoods. This approach will provide a robust assessment of the economic burden on communities on both sides of the international border. The planned statistical analyses include parametric *t*-tests to compare the average values of parameters, fixed effect/random effect-panel/pooled OLS regression to find out the determinants of variation in health outcomes, Structural Equation Modeling to disentangle the direct impact of socioeconomic conditions on health outcomes and the effect of socioeconomic conditions on health outcomes, mediated by exposure to the ambient air pollution, and Density Curve analysis and Propensity Score Matching to see the difference in effect on productivity and wage loss among groups in the upwind and downwind sites. Theoretical frameworks such as health-related productivity loss (HRPL) and loss of human capital will be utilized to understand the economic burden of exposure to pollution. Data collected on medical expenditure, loss of workdays of the patient and the accompanying persons, and their nature of occupation, combined with the prevailing market wage, would provide a nuanced understanding of the loss of productivity, economic burden of health expenditure, and loss of human capital. A vulnerability index could be developed based on socioeconomic conditions, exposure to outdoor activities, smoking habits, prevailing health conditions (such as asthma), and additional exposure to indoor air pollution due to biomass burning.

**Table 1:** Schematic of the study questionnaire for gathering key information in the investigation.

**Section 1. Demographic characteristics of household members**
i. Name, age, and gender
ii. Who is the household head?
iii. Marital status
iv. Number of years of their schooling
v. Occupation type
vi. Hours spent cooking indoors
**Section 2. Socio-economic characteristics of the household**
i. Long-run worries in their household/locality
ii. Perception about the outdoor air quality and their sources
iii. Type of housing and brief details about their residence
iv. Primary source of drinking water
v. Status of electricity connection
vi. Fuel types and stoves used for cooking
vii. Type of ventilation in the kitchen
viii. Assets owned
ix. Total expenditure every month and income status
x. The domain in which they are willing to invest if income increases by 10% (e.g., housing, education, civic amenities, etc.)
**Section 3. Health characteristics of every individual in the household**
**3.a. Morbidity and smoking habits**
i. Heredity disease prevalence and status of medications
ii. Details on the consumption of tobacco-based products
**3.b. Morbidity without hospitalization in a recall period of**
**three months**
i. Health-related symptoms with frequency, severity, cost incurred, wage, and work day lost
**3.c. Morbidity with hospitalization with a recall period of**
**three months**
i. Reason and number of days for hospitalization
ii. Type of hospital
iii. Cost incurred during the hospitalization
iv. Loss in wage and work days due to hospitalization
v. Status of insurance or health scheme, if available
**3.d. Mortality in the last 365 days**
i. Name, Age, and Gender of the deceased member
ii. Details of hospitalization before the mortality
iii. The status and details of any chronic ailment, if present
iv. Was he/she a primary earning member, and their occupation

### 3.3 Chemical speciation, tracers, and source apportionment of pollutants

Time-integrated (24 h) aerosol sampling was conducted using a combination of high- and low-volume PM_2.5_ samplers on a 24 h basis during winter, summer, monsoon, and post-monsoon of 2021–2022 at the Indian *Case* and *Control* sites, and during winter 2022–2023 at the sites in Bangladesh. In Bangladesh, only winter sampling was conducted based on the nature of the plume's dispersion. Meteorological data (temperature, wind speed, wind direction, relative humidity, precipitation) were recorded for the entire sampling period. The PM samples are being processed targeting comprehensive screening of inorganic and organic chemical markers for combustion and health-related impacts (e.g., ionic species, organic and elemental carbon, potentially toxic elements, black carbon, polycyclic aromatic hydrocarbons, n-alkanes, humic substances, and levoglucosan). Based on the receptor model, Positive Matrix Factorization (PMF), this dataset will be used to investigate source contributions. The model will provide quantitative estimates of contributions from various sources (e.g., dust, vehicular and industrial emissions, and biomass burning) and the TPP, influencing air quality and respiratory health. Furthermore, we will measure the generation of reactive oxygen species (ROS) with the acellular dithiothreitol (DTT) assay (17), and correlate PM composition and its oxidative potential with lung status at different sites, aiding in predicting lung health outcomes.

### 3.4 Tracing the impacts on respiratory health and biological aging

Participant recruitment and consent procedures were followed, after which adults underwent lung function tests, including spirometry and FeNO measurements (Figure 2). Abnormalities in the subject's lung health could be due to inflammation and small and large airways narrowing from PM exposure (18). The narrowing of airways is measured by spirometry [forced expiratory volume in 1 s (FEV_1_), forced vital capacity (FVC), and forced expiratory flow between 25% and 75% of vital capacity (FEF_25 − 75_)], while FeNO measures airway inflammation. FeNO measurements will also be used as a Type 2 inflammation biomarker, representing a significant asthma phenotype with overlapping relevance in COPD phenotypes (19). The spirometry method meets the ATS-ERS criteria of acceptability and reproducibility. Abnormalities in the subjects would be classified as obstructive, restrictive, small airway disease, and preserved ratio impaired spirometry (PRISm) (20). Also, blood samples were collected that will be analyzed for pulmonary-induced systemic inflammation and the development of clinical biomarkers specific for respiratory complications and biological aging from chronic exposure. The respondents' ages will be defined in transcriptomics aging clocks as biological and compared with the subjects' chronological age. The biological age will be estimated using a molecular clock based on gene expression data using transcriptomics age estimators such as BitAge (21), BURNS (22), and RNAAgeCalc (23). In addition, we will calculate Δ_age_ by subtracting the chronological age from the transcriptomic age. The Δ_age_ will be used to categorize the subjects into three groups: people with accelerated aging (Δ >5), regular (−5 < Δ <5), and a declining aging process (Δ < −5) (24). These tests will provide quantitative and comprehensive insights into biological vs. cellular aging influenced by environmental conditions, lifestyle, and medication (24, 25). Lastly, using an advanced network-based integration analysis, we will evaluate the data on how aerosols and socioeconomic status affect biological aging (26, 27).

## 4 Quality control, data management, and communication

Each parameter measured or estimated in this study will follow QA/QC protocols, e.g., organic/inorganic tracers will be assessed using calibration curves (*R*^2^), recovery of spiked standards, comparison with NIST SRMs, repeatability, and detection limits; lung function tests were conducted based on globally accepted protocols, etc.

Linköping University coordinates the study and oversees data management, quality assurance, and control. Partner institutions in India, Bangladesh, and Sweden jointly lead field surveys, data collection, and laboratory analyses. Personal data will be handled according to national regulations, with only pseudo-anonymized data transferred to Sweden; identification keys will remain in the respective countries. Chemical and model outputs will be shared through open-access platforms such as the AQI, EEA AirBase, and the Air Quality Global Open Database. All data will be securely stored on Linköping University's servers, following institutional data stewardship policies. The communication Divisions at Linköping University and Karolinska Institute will support communication using SMART principles across media, newsletters, and web platforms.

## 5 Study limitations and strengths

The primary limitations of this study include logistical challenges associated with, e.g., working in remote areas, the time-intensive nature of field and laboratory analyses, and sample preservation in the field. Changing wind patterns require additional high-volume samplers to ensure robust data collection. However, financial constraints and the significant workforce needed for maintenance and pre- and post-sampling processes make this problematic. Additionally, there is a risk of loss in following up among participants over the study period (e.g., sick, away from home, death, etc.), which could stall data collection and impact the results.

Studies investigating lung functionality are typically conducted in hospital settings. The strength of this study lies in the health team's active involvement in conducting lung tests directly at the household level. This approach offers a comprehensive snapshot of the current air pollution status and helps establish causality by providing a clear spatiotemporal trend between pollution levels, exposure, and health outcomes.

## 6 Potential outcomes

This interdisciplinary prospective study provides a systematic framework to investigate the energy-health-environment nexus in areas affected by TPP emissions. Potential outcomes include: 1) aerosol modeling of the PM plume and its geographical footprint, subject to current atmospheric conditions and dispersion pattern, 2) chemical characteristics of PM, including source apportionment and estimation of oxidative potential, and 3) the influence of socioeconomic conditions on health resulting in widespread respiratory ailments and accelerated lung-aging. In particular, ongoing investigations and preliminary model outputs demonstrate that PM emissions from the TPP extend tens of kilometers downwind, highlighting a growing transboundary health and economic burden on the community living in this area. Addressing these complex and interrelated issues demands regional collaboration and a robust action plan to reduce the impacts on air quality and people's health in both India and Bangladesh following detailed surveys, negotiations, and strategic interventions to 1) “phase down” the dependence on coal and contain the damage to people's lives and livelihoods, and 2) address the historical legacy of handling environmental pollution from coal-fired TPPs. Such coordinated actions are essential for fostering sustainable solutions that balance these countries' environmental health and socioeconomic wellbeing across borders.

## Data Availability

The original contributions presented in the study are included in the article/supplementary material, further inquiries can be directed to the corresponding authors.
